# Validation and Application of a Custom-Designed Targeted Next-Generation Sequencing Panel for the Diagnostic Mutational Profiling of Solid Tumors

**DOI:** 10.1371/journal.pone.0154038

**Published:** 2016-04-21

**Authors:** Guy Froyen, An Broekmans, Femke Hillen, Karin Pat, Ruth Achten, Jeroen Mebis, Jean-Luc Rummens, Johan Willemse, Brigitte Maes

**Affiliations:** 1 Department of Clinical Biology, Jessa Hospital, Hasselt, Belgium; 2 Department of Pneumology, Jessa Hospital, Hasselt, Belgium; 3 Department of Pathology, Jessa Hospital, Hasselt, Belgium; 4 Department of Medical Oncology, Jessa Hospital, Hasselt, Belgium; 5 Department of Clinical Biology, AZ Turnhout, Turnhout, Belgium; University of Verona, ITALY

## Abstract

The inevitable switch from standard molecular methods to next-generation sequencing for the molecular profiling of tumors is challenging for most diagnostic laboratories. However, fixed validation criteria for diagnostic accreditation are not in place because of the great variability in methods and aims. Here, we describe the validation of a custom panel of hotspots in 24 genes for the detection of somatic mutations in non-small cell lung carcinoma, colorectal carcinoma and malignant melanoma starting from FFPE sections, using 14, 36 and 5 cases, respectively. The targeted hotspots were selected for their present or future clinical relevance in solid tumor types. The target regions were enriched with the TruSeq approach starting from limited amounts of DNA. Cost effective sequencing of 12 pooled libraries was done using a micro flow cell on the MiSeq and subsequent data analysis with MiSeqReporter and VariantStudio. The entire workflow was diagnostically validated showing a robust performance with maximal sensitivity and specificity using as thresholds a variant allele frequency >5% and a minimal amplicon coverage of 300. We implemented this method through the analysis of 150 routine diagnostic samples and identified clinically relevant mutations in 16 genes including *KRAS* (32%), *TP53* (32%), *BRAF* (12%), *APC* (11%), *EGFR* (8%) and *NRAS* (5%). Importantly, the highest success rate was obtained when using also the low quality DNA samples. In conclusion, we provide a workflow for the validation of targeted NGS by a custom-designed pan-solid tumor panel in a molecular diagnostic lab and demonstrate its robustness in a clinical setting.

## Introduction

Targeted therapies for solid tumors have shown great promise and based on ongoing clinical studies, the potential to enlarge the current set of approved anti-cancer drugs is huge [1,2]. As most of these drugs target specific signaling pathways it is of outmost importance to detect mutations in the critical genes involved allowing for precision medicine [3,4].

Currently, most diagnostic labs are applying standard molecular technologies (qPCR, melt curve analysis, Sanger and pyrosequencing, etc.) to screen solid tumor samples for the presence of a selected number of actionable hotspot mutations, mainly in *BRAF*, *EGFR*, *KRAS*, *NRAS*, *KIT* and *PIK3CA*. Although these methods are well installed and highly reliable, they require a significant amount of DNA since the analysis of each gene needs a specific assay. This amount is often restricted due to the limited availability of tumor tissue. Moreover, the turn-around time (TAT) from tumor resection to results, estimated at 1 up to 4 weeks, is even prolonged since subsequent analysis of different hotspots with different assays is often required [5]. Therefore, the standard assays are currently being replaced by multiplex parallel analysis *i*.*e*. next-generation sequencing (NGS).

NGS-based diagnostic screening for tumor-associated mutations using whole genome or exome sequencing is currently not preferred because of the high cost, the bioinformatics’ challenge, the need of high depth of coverage when sequencing diagnostic tumor samples, manage and store large data amounts, and the question how to handle incidental findings. For now, sequencing of targeted panels is much more favorable for diagnostic laboratories. The selected diagnostic, prognostic and actionable hotspots can be screened simultaneously thereby requiring low amounts of DNA, generate high depth of coverages and allow a fast TAT at a reasonable price per sample. Though targeted panels can consist of hundreds of genes [6,7] they mostly constitute a much smaller set typically between 20 and 50 genes, providing information on 50 to 250 hotspots [8–10]. As a consequence, targeted assays make the run, analysis and interpretation much faster, easier and cheaper without compromising on its associated clinical value [4,11,12].

It is currently not feasible to install fixed rules to which molecular laboratories have to adhere in order to acquire diagnostic accreditation for clinical oncology mainly because of the lack of uniformity throughout the entire NGS process [13]. However, guidelines for diagnostic validation have been published by several groups [14–17], but these are often generalized or focused on specific topics of the NGS pipeline [18]. In any case, a diagnostic lab should prove its ability to accurately detect and report the different types of mutations, with the aim to guide personalized medicine in relation to the patients’ tumor.

Here, we report on the validation and clinical implementation of targeted NGS for the analysis of hotspot mutations in FFPE sections from non-small cell lung carcinoma (NSCLC), colorectal carcinoma (CRC) and malignant melanoma (MELA). We selected hotspot regions in 24 genes, enriched those with the TruSeq method and sequenced library pools of 12 samples per MiSeq run. Our validation demonstrated its applicability in molecular profiling with subsequent implementation in routine diagnostics.

## Materials and Methods

### Patient Samples and DNA Extraction

The committee of Medical Ethics of the Jessa Hospital (Hasselt, Belgium) has approved this study. According to the Belgian law of 19 December 2008 with number N. 2008–4682 [C– 2008/18385], no written informed consent is required. The ethical committee has thus waived the need for written informed consent from the patients.

Resections or biopsies of solid tumors, from the primary or from a metastatic site, were taken and sent to the pathologists for review. About 80% and 50% of NSCLC and CRC samples, respectively were biopsies. Formalin-fixed paraffin-embedded (FFPE) blocks were cut into 5 μm thick tissue sections for pathological and molecular analysis in the order: first H&E stained slide; 5 slides for (immuno)histological analysis, second H&E stained slide; 7 slides for DNA extraction; third H&E stained slide; 3 slides for DNA extraction; 2 slides for FISH. Tumor regions were marked on the third hematoxylin and eosin (H&E)-stained section. In case of an estimated number of >100 tumor cells and a tumor content ≥10%, the corresponding regions were scraped from 5 to 10 unstained serial sections and DNA was extracted with the QiaSymphony DNA mini kit on a QiaSymphony robot (Qiagen, Hilden, Germany). DNA was eluted in 50 μl ATE buffer and stored frozen at -20°C. The DNA concentration was quantified with the Qubit dsDNA BR assay kit on a Qubit fluorometer 3.0 (Lifetechnologies, Carlsbad, CA). The Quantitation multiplex reference standard HD701 (Horizon Dx, Cambridge, UK) that contains 11 clinically relevant mutations in 6 genes at known variant allele frequencies (VAF) from 1% to 25%, was used as a reference sample to test the accuracy of the NGS workflow.

### Analysis of QC Value

To maximize the efficiency of NGS on FFPE DNA samples it is recommended to assess the quantity and quality of these samples. This analysis was performed by an in-house developed duplex qPCR that amplifies fragments of two different lengths, a short 47 bp to assess DNA quantity, and a 268 bp amplicon for DNA quality (i.e. modification and degradation) measurement. The QC value is measured as the delta threshold cycle (dCt) relative to the quality control template (QCT) of the FFPE QC kit from Illumina (San Diego, CA). Our in-house developed assay was compared with this commercial FFPE QC kit on 20 samples. Primer sequences can be obtained upon request. As recommended by the Illumina kit, the qPCR was performed on 1/100 dilutions of the DNA samples. The reactions were done in a total of 15 μl containing 1x Absolute qPCR SYBR green mix (AbGene, Portsmouth, NH), and 250 nM of the primer mixture on a RotorGene Q instrument (Qiagen) with the annealing temperature at 60°C. The dCt (Ct sample—Ct reference) was then calculated for each sample. The lower the dCt is, the better the QC value of that sample.

### Custom Tumor Gene Panel

For the design of the panel content, the primary aim was to allow for the detection of actionable mutations that is at present required in Belgium for selection of NSCLC, CRC and MELA patients for respectively anti-EGFR tyrosine kinase inhibitors, anti-EGFR monoclonal antibodies and anti-BRAF therapy and to replace the conventional methods. In addition, the panel content should allow to detect other potential targets for therapies that might become available in the future or mutations with diagnostic or prognostic significance, in several solid tumor types. We aim to use this custom panel as a pan-solid tumor panel for diagnostic molecular profiling of all solid tumor types.

Based on the gene content of commercial cancer panels, the literature and databases including My Cancer Genome (http://www.mycancergenome.org/) and PCT MD Anderson (https://pct.mdanderson.org/#/) we composed a predefined list of recurrent, pathogenic mutations in 16 oncogenes. In addition, frequently altered regions in 8 tumor suppressor genes, resulting in loss-of-function mutations, were selected. The final content consisted of a target region in 24 genes including *AKT1*, *ALK*, *APC*, *BRAF*, *CDKN2A*, *CTNNB1*, *EGFR*, *ERBB2 (HER2)*, *FBXW7*, *FGFR2*, *GNA11*, *GNAQ*, *KIT*, *KRAS*, *MAP2K1*, *MET*, *NRAS*, *PDGFRA*, *PIK3CA*, *PTEN*, *RET*, *SMAD4*, *STK11* and *TP53*. The transcript ID numbers as well as the solid tumor hotspots within these genes are provided in S1 Table. The probes for this targeted custom panel were designed with DesignStudio (Illumina) and consisted of 120 amplicons with an average size of 150 bp and a cumulative targeted region of 8.1 kb. Polymorphisms were avoided in the design of primers. Hotspots of recurrent mutations were not located in primer sites either.

### Targeted NGS

Target enrichment was performed on FFPE-extracted DNA using the protocol described in the TruSeq Amplicon—Cancer Panel Library Preparation Guide (April 2014; Illumina). In brief, two probes per targeted amplicon are hybridized to the same strand of the patient’s genomic DNA after which the 120–180 bp regions in between the probe pairs are filled up and ligated. After removal of unbound probes, the produced targeted regions are PCR-amplified using two universal primers each containing an 8-mer index for patient tagging. Since the maximal volume that can be added in the capture is 15 μl the total amount of input DNA for capture ranged from <10 ng (for a DNA sample with the DNA concentration below the detection limit) to 250 ng. Twelve samples were processed simultaneously. After library preparation, indexing and bead purification, the libraries were quantified by Qubit analysis to assess successful enrichment and amplification. Libraries were then normalized on beads and pooled for sequencing according to the TruSeq Amplicon protocol. The pooled libraries were paired-end (2x151) sequenced on a micro flow cell with V2 chemistry on a MiSeq instrument (Illumina). The PhiX control library (Illumina) was spiked in each run at a final concentration of 12 pM to estimate the sequencing error rate, as described in the manufacturer’s protocol.

### Data Analysis and Classification of Variants

**Fig 1:**
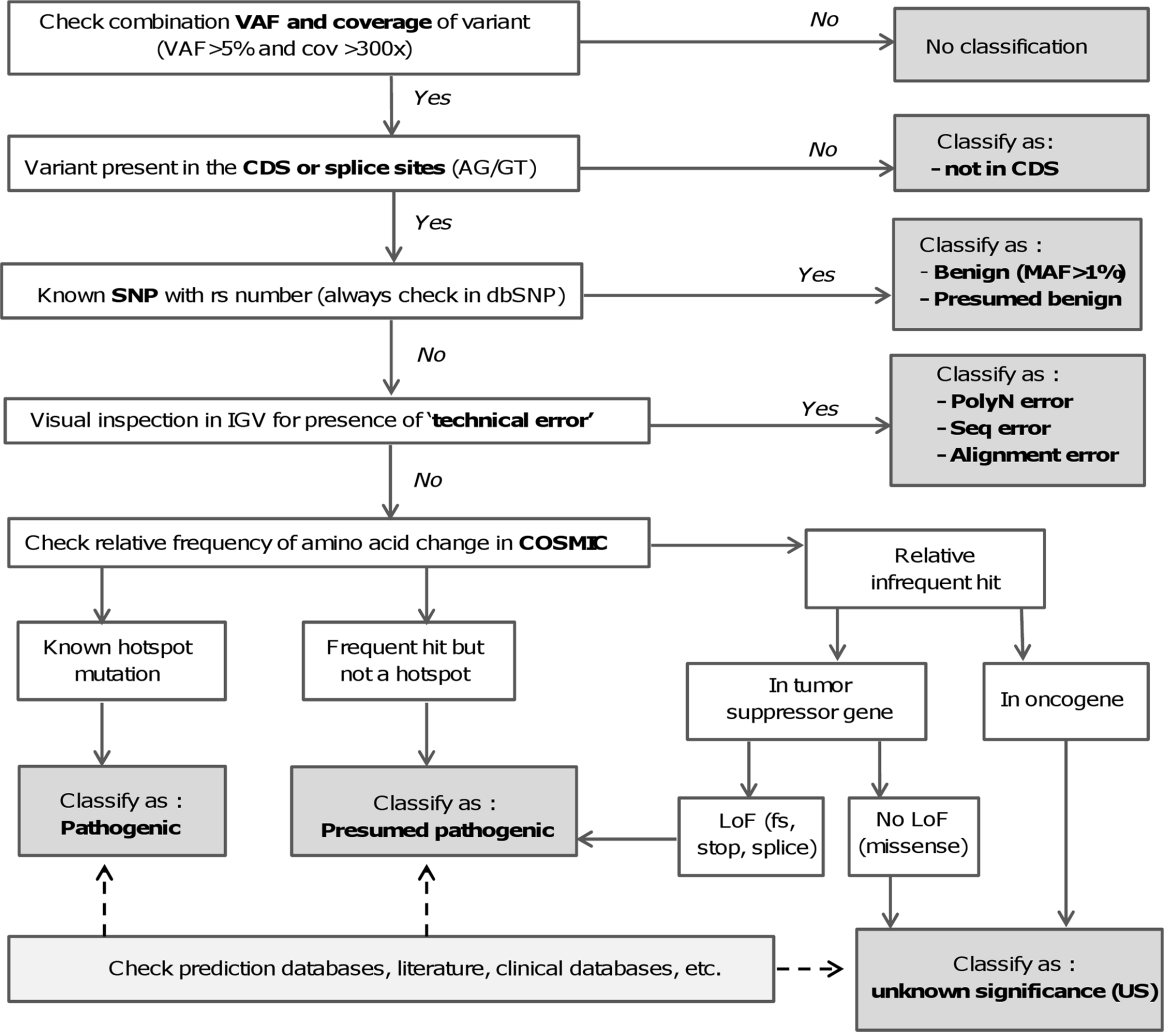
Flow chart for variant analysis. This chart is used for our classification of variants in solid tumor samples. VAF: Variant allele frequency; CDS: coding sequence; SNP: single nucleotide polymorphism; MAF: Minor allele frequency; IGV: Integrative Genomics Viewer; LoF: Loss-of-function.

The data were automatically analyzed with the MiSeqReporter software (Illumina) on the instrument using the Targeted Resequencing—TruSeq Amplicon settings, which generate the nucleotide sequences with their base quality scores in text format file (Fastq), the Binary alignment/map file (Bam) and Variant calling file (Vcf) for each individual sample. Alignment was done against the human reference sequence build GRCh37/Hg19. Patients’ anonymous data were saved on the instrument as well as in the cloud (BaseSpace). The Bam files were loaded in Integrative Genomics Viewer (IGV; The Broad Institute, Cambridge, MA) for read visualization and the Vcf files were imported in VariantStudio (Illumina) for variant annotation and filtering. The classification was done based on our in-house built flow chart (Fig 1) and stored in the database of VariantStudio. Predefined pathogenic mutations were annotated as such at their first encounter. For other variants, consisting of novel exonic non-synonymous and frameshift variants as well as intronic splicing variants the COSMIC database (http://cancer.sanger.ac.uk/cosmic) was consulted for the classification as either pathogenic, presumed pathogenic or ‘variant of unknown significance’ (Fig 1). Finally, all hotspots were automatically analyzed for sufficient coverage (validated at a cut-off of 300) using an in-house-developed macro in Excel (Microsoft) applied on the coverage.tsv file located in the analysis folder of each run.

### Standard Diagnostic Assays

We employed our ISO 15189:2012 certified orthogonal diagnostic tests for solid tumors to check and confirm the NGS data. These include the Therascreen *EGFR* RGQ PCR kit (Qiagen) that detects 29 somatic mutations in exons 18 to 21 of *EGFR*, and the Therascreen *BRAF* RGQ PCR kit (Qiagen) for analysis of five somatic mutations at amino acid V600 of *BRAF*. For RAS screening, we used an in-house developed *KRAS* G12-G13 qPCR to screen for 7 *KRAS* mutations in codons 12 and 13, complemented with pyrosequencing on the PyroMark Q24 (Qiagen) with the Therascreen *RAS* extension Pyro kit (Qiagen) for detection of mutations in *KRAS* codons 59, 61, 117 and 146. With the same method, mutations in codons 12, 13, 59, 61, 117 and 146 of *NRAS* were screened for by the Therascreen *NRAS* Pyro kit (Qiagen).

## Results

We performed the validation of the targeted NGS workflow on 55 FFPE samples (14 NSCLC, 36 CRC, 5 MELA) as well as the Multiplex reference sample. These samples were used to assess the repeatability (intrarun: 6 samples), reproducibility (interrun: 6 samples; interoperator: 4 samples), accuracy (reference sample), limit-of-detection (4 samples), and analytical sensitivity and specificity (40 samples). An overview of the samples used for each assay can be found in S2 Table. From these validation experiments, we defined the minimal coverage and variant allele frequency (VAF) required to correctly call a variant. Next, 150 diagnostic samples (99 NSCLC, 40 CRC, 11 MELA) were analyzed.

### Sequence Coverage

To assess the sequencing quality at each hotspot position we used the coverage per amplicon and the mean coverage per sample as proxies. Both coverage values are automatically generated for every sample in the run via an in-house developed macro-mediated coverage file (S3 Table). For all samples we check the coverage at each amplicon, which corresponds to the coverage of a hotspot present in that amplicon. Based on our validation data of the 55 samples, we defined a minimal amplicon coverage of 300 and a variant allele frequency (VAF) of 5% as the minimal thresholds to provide reliable diagnostic analysis of solid tumor samples. These cut-offs were chosen based on the absence of false positive or false negative variants above these combined thresholds (see sections ‘Accuracy‘ and ‘Analytical sensitivity and specificity‘) for any tested position, including single nucleotide variants (SNVs) and short insertions and deletions (indels). Variants in hotspots with a coverage <300 were regarded as non-informative. For those ‘failed’ hotspots the orthogonal assay for the requested targets or a repeat NGS analysis will always be performed. The failed hotspots are listed for each sample in the row ‘hotspots in failed amplicons’ in the coverage file (S3 Table). We noticed a high variability in coverage between different amplicons. The BRAF V600-containing amplicon was enriched and/or sequenced much more abundantly than the EGFR G719-carrying amplicon. Therefore, the successful analysis of the BRAF hotspot will be much higher when compared to the EGFR G719 position. The amplicons that most often fail are CDKN2A R58,R80 and STK11 F354 (S3 Table).

The mean coverage of all target regions of each sample can additionally be used to assess its enrichment and/or sequencing quality. Samples with a low mean coverage will always present with many failed amplicons and thus will not provide robust data. Again, based on our validation data we have set the minimal threshold for the mean coverage of a sample at 300. Samples that do not reach this threshold are regarded as unreliable and these will not be further analyzed. Samples with a mean coverage between 300 and 800 will be further analyzed but only for the requested hotspots relevant for the histological subtype (eg. *EGFR* in NSCLC). Finally, samples with a mean coverage >800 will be analyzed for all hotspots. However, individual failed hotspots (coverage <300) will be listed in the report as non-informative. Based on these settings we analyzed 150 diagnostic samples with NGS and found20 (13%) with an insufficient coverage (<300) and nine (6%) a coverage between 300 and 800 reads.

The minimal VAF threshold was set to 5%. However, variants with a VAF <5% are not *a priori* disregarded. For a variant with well-known pathogenicity and actionability, the combination of VAF and coverage has to be judged carefully since the higher the coverage, the more likely it is that the variant is a true mutation. The NGS analysis is then repeated (on another sample if available) or the orthogonal assay for that variant is performed.

**Fig 2:**
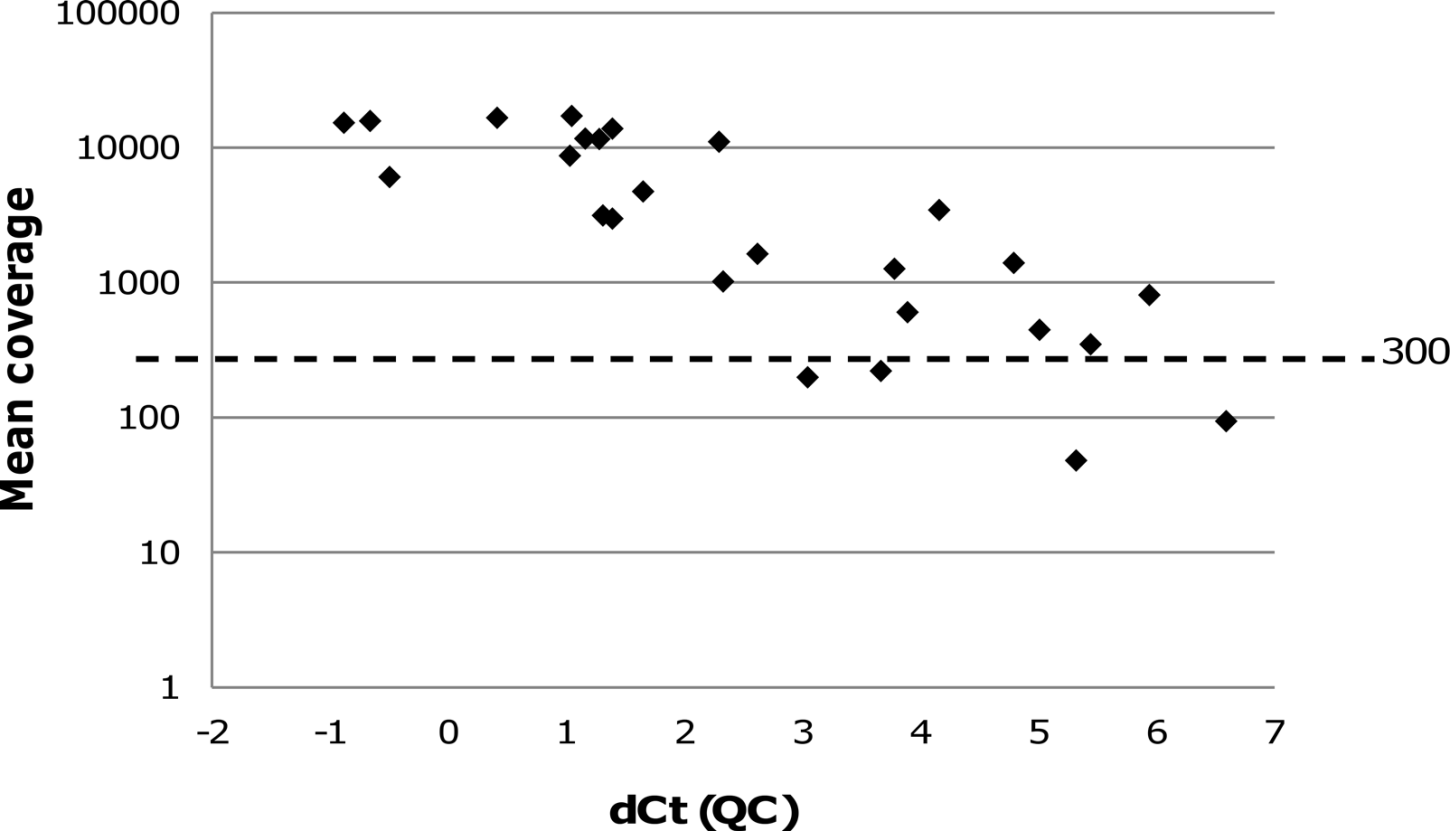
Correlation of dCt with mean coverage. Plot showing the correlation of the DNA quality (QC), expressed as dCt, with the mean coverage (log scale) of 26 random FFPE validation samples.

To assess whether the input amount of DNA in the capture step correlates with the sequencing quality we plotted the association of the DNA amount and the mean coverage for the 55 FFPE validation samples. Interestingly, no correlation at all was found (S1 Fig). Samples with input amounts <50 ng had highly variable mean coverages ranging from 319 to 7477. Also, the samples with a mean coverage <300 did not have the lowest DNA input amounts. Moreover, plotting the QC value, determined as the dCt of the qPCR assay, against the mean coverage of 26 random FFPE validation samples showed no absolute correlation either, especially also not for samples with less good quality (dCt>2) (Fig 2). Finally, we plotted the estimated tumor zone size of the FFPE sections against their obtained mean coverage and again, demonstrated that the tumor zone size is not an absolute predictor of the mean coverage for any of the three tumor types (S2 Fig). These data, combined with the fact that an additional qPCR will result in a delay of the turnaround time, we decided not to exclude any specimen from NGS analysis.

### Repeatability and Reproducibility

The repeatability (intra-run) was tested on 6 samples (3 NSCLC, 2 CRC and 1 MELA) with known mutations, of which four were analyzed in duplicate and two in triplicate. Different indices were always used for matched samples. The twelve mutations, including single nucleotide variants (SNVs) and indels **(**S4 Table**),** were detected in the replicate samples even though one sample (CRC2) had an insufficient number of reads. Importantly, the identified VAFs per sample were highly similar as indicated by the SDs. To test the reproducibility (inter-run), we analyzed 6 FFPE samples (1 NSCLC, 4 CRC and 1 MELA) in different runs. Again, our data demonstrated high reproducibility for missense mutations and indels (S5 Table**)**. Finally, the inter-operator variability was tested for 4 samples (1 NSCLC, 3 CRC) and demonstrated equally good reproducibility with VAFs of similar percentages (S6 Table). Since SNVs as well as subtle deletions and insertions were included for multiple positions in several genes in the three tumor types with variable VAFs, we can conclude that the method is highly reproducible for the detection of mutations in FFPE samples. An overview of the number and type of samples tested can be found in S2 Table.

### Accuracy

The accuracy of the assay to determine the analytical performance was tested on the Multiplex reference standard starting from 100 ng and 200 ng DNA. For both samples, all 11 known variants were detected at highly similar VAFs (Table 1). Moreover, three additional variants in *MAP2K1* and *CTNNB1* were also found at somatic VAFs in both samples. As the detected VAFs very well matched those reported by the company, our data strongly suggest that the VAFs that we obtain in our clinical samples are representative for the true allele frequencies. Secondly, the accuracy was also assayed by the NGS data of the FFPE samples employed for the other validation tests, including SNVs and indels as well as those with VAFs close to the limit of detection (S2 Table).

**Table 1:** The accuracy of the NGS workflow was checked with the Horizon Quantitation multiplex reference that contains 11 mutations at known variant allele frequencies (VAF). NGS data are provided for DNA start amounts of 100 ng and 200 ng. Mean and standard deviations (SD) are given as well. del: deletion; nr: not reported.

Gene	Mutation	Horizon Dx VAF(%)	NGS 200 ngVAF(%)	NGS 100 ng VAF(%)	Mean NGS (%) (SD) (%)
			
*BRAF*	V600E	10.5	10.7	10.8	10.8 (0.1)
*KIT*	D816V	10.0	10.0	10.8	10.4 (0.5)
*EGFR*	ex19del12bp	2.0	2.0	2.5	2.2 (0.3)
*EGFR*	L858R	3.0	3.2	3.0	3.1 (0.1)
*EGFR*	T790M	1.0	1.0	1.0	1.0 (0.0)
*EGFR*	G719S	24.5	26.4	27.4	26.9 (0.7)
*KRAS*	G12D	15.0	16.2	13.2	14.7 (2.2)
*KRAS*	G12D	6.0	5.8	8.8	7.3 (2.1)
*NRAS*	Q61Q	12.5	14.4	15.3	14.8 (0.6)
*PIK3CA*	H1047R	17.5	16.8	18.5	17.6 (1.2)
*PIK3CA*	E545K	9.0	8.9	10.9	9.9 (1.4)
*MAP2K1*	Q56P	nr	33.3	32.5	32.9 (0.6)
*MAP2K1*	S33Y	nr	31.4	32.1	31.8 (0.5)
*CTNNB1*	S45del	nr	11.0	9.1	10.0 (1.3)

### Analytical Sensitivity and Specificity

We have screened 40 prospective samples (11 NSCLC, 27 CRC, 2 MELA; S2 Table) with NGS as well as with the relevant orthogonal assay for the actionable mutations in *BRAF*, *EGFR*, *KRAS* and *NRAS*. In 20 samples, a mutation was detected with targeted NGS (coverage >300 and VAF >5%) and confirmed by the standard assay (S7 Table). Therefore, no false positives were obtained by NGS yielding a maximal specificity. The mutations included 10 different SNVs and a 9 bp insertion in exon 20 of *EGFR*. In four of the samples however, NGS detected variants not confirmed by the standard method but all four did not meet the acceptance criteria and thus were correctly excluded. The errors were detected in *EGFR* (G719V; VAF 63%, coverage 145, and G719S; 27%, 215) and *NRAS* (G12D; 2%, 913 and A146V; 16%, 109). Variants detected by NGS in other genes are not shown because we do not have a standard assay available for their confirmation. However, for the samples that were also used to assess the repeatability or reproducibility all ‘other’ variants were confirmed by the replicates (S4–S6 Tables).

Similarly, the remaining 20 prospective samples in which no actionable mutation in the four genes was detected by NGS, did also not reveal a mutation with the standard diagnostic assays demonstrating absence of false negatives by NGS and thus a maximal sensitivity.

### Limit-of-Detection

**Fig 3:**
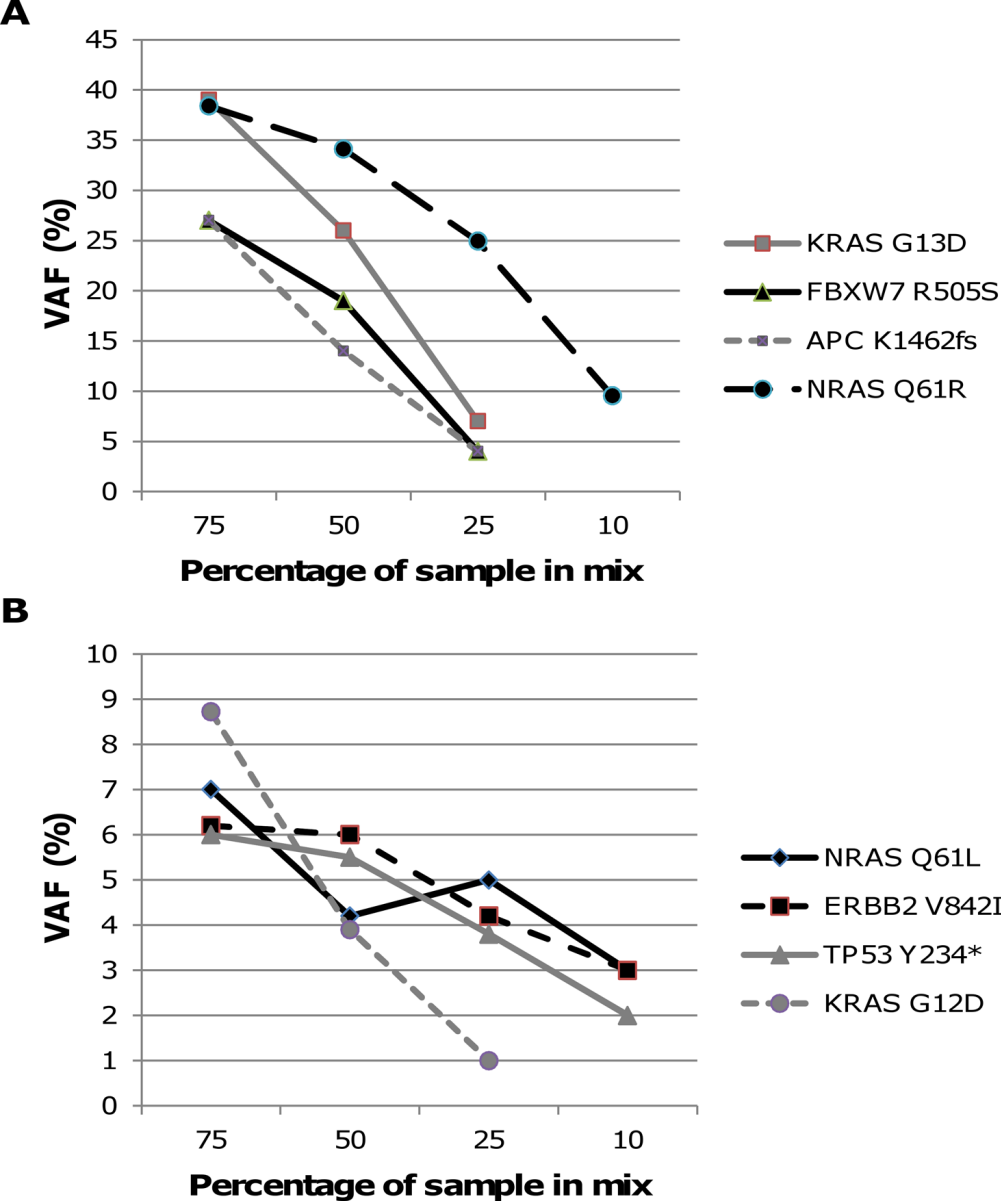
Limit-of-detection assay. Two samples with known mutations were mixed at different percentages, captured and sequenced. VAFs of each mutation were plotted against the percentage in the mix. (A) Mutations KRAS G13D (58%), FBXW7 R505S 48%), APC K1462fs (52%), NRAS Q61R (35%) with high VAFs in the original sample. (B) Mutations NRAS Q61L (9%), ERBB2 V842I (8%), TP53 Y234* (9%), KRAS D12D (24%) with low VAFs in the original sample.

Previous analysis of the Horizon reference standard confirmed all variants including SNVs and indels up to 2–3% VAF (Table 1). The *EGFR* T790M mutation at VAF 1% was not detected via MiSeqReporter but its occurrence at 1% was confirmed in the IGV read viewer. To assess the lower detection limit of mutations in FFPE samples we made variable mixes of two times two DNA samples (1 NSCLC, 2 CRC, 1 MELA) with two known mutations each, to reach final sample contributions in the mixes of 75%, 50%, 25% and 10%. After capture and sequencing, the lowest detectable VAF for each mutation was determined. As expected, the VAF for all 8 mutations decreased with a decreasing contribution of the sample in the mix (Fig 3). For mutations with a high initial VAF in the original sample (Fig 3A) the lowest VAF reached down to 5% because of the restricted dilution of these samples. However, for mutations with lower initial VAFs (Fig 3B) the tested variants could efficiently be detected as low as 1–3%. Notwithstanding this very low limit of detection we have set the VAF minimal threshold at 5% for robust diagnostic screening. For samples that had significant deamination the VAF threshold was set to 10% for C>T or G>A changes. DNA deamination was evident from the co-occurrence of a more extensive variant list with the predominant presence of C>T or G>A changes (>50%). Deamination was exclusively found in FFPE samples stored for at least one year.

### NGS QC Measures

Monitoring the quality of the different steps during the NGS workflow is required in order to obtain accurate and reliable patient results. Therefore, we inspect and log several sample and run parameters. First, the efficiency of the target enrichment and library preparation is analyzed through Qubit quantification of the purified libraries. Failures in one of the enrichment steps will result in concentrations below detection for all samples, which serve as a hard stop in the workflow. To monitor the run quality we have set thresholds for the following parameters: cluster density >500 K/mm^2^, cluster pass filter >70%, Q30 >80%, and error rate <1.2% (based on the PhiX spike-in). The data of these parameters for 10 representative diagnostic runs are provided in S8 Table and all show passing values with low variability. As expected, runs with higher cluster densities (>1400) had generally lower cluster pass filter, poorer %≥Q30 scores and higher error rates. Finally, the mean coverage per sample and the coverage at each single hotspot are inspected via an in-house developed macro (S3 Table).

### Implementation in Diagnostics

After the successful validation process we diagnostically sequenced 150 FFPE samples from CRC (40), NSCLC (99), and MELA (11) origin (Table 2) that had not been previously used for the validation assays. Of these, an insufficient number of reads (mean coverage <300) was obtained for 20 (13%) samples (4 CRC and 16 NSCLC), which was not always due to a low start amount of DNA (12 samples <50 ng). Furthermore, a low input amount did not always correspond with poor performance as 32 samples with an input <50 ng yielded a mean coverage >300 of which 20 even had >1000 coverage. In 29 (22%) from the 130 samples with a sufficient mean coverage (>300), we did not find any pathogenic or presumed pathogenic mutation with a VAF >5%. In the other 101 specimens, a total of 155 pathogenic or presumed pathogenic variants were detected in 16 genes (Table 2). The most abundant mutations in a therapeutically important gene were present in *KRAS* (42/130: 32%) followed by *BRAF* (12%), *EGFR* (8%) and *NRAS* (5%). *KRAS*, *NRAS* and *BRAF* mutations were detected in two or all three tumor types while mutations in *EGFR* were uniquely detected in NSCLC. In the genes with diagnostic or prognostic value, loss-of-function mutations in *TP53* were most prevalent (32%) detected in all three tumor types, followed by deleterious mutations in the tumor suppressor genes *APC*, *FBXW7* and *SMAD4* in CRC, and *CDKN2A* in MELA.

**Table 2:** Targeted NGS analysis on 150 diagnostic FFPE samples. Number (#) and percentages (%) of samples and variants are indicated for the three tumor types investigated. Only pathogenic or presumed pathogenic variants that met the acceptance criteria (Cov >300; VAF >5%) were included. Many samples had more than one mutation. Insuff reads: mean coverage <300. total #: total number of variants.

	CRC		NSCLC		MELA		total #
	#	%	#	%	#	%	or %
# samples	40	100%	99	100%	11	100%	150
insuff reads	4	10%	16	16%	0	0%	20 (13%)
remaining	36	90%	83	84%	11	100%	130
no variants detected	5	14%	22	22%	2	18%	22%
BRAF	5	14%	8	10%	3	27%	12%
EGFR	0	0%	10	12%	0	0%	8%
KRAS	15	42%	27	33%	0	0%	32%
NRAS	0	0%	4	5%	3	27%	5%
AKT1	1	3%	0	0%	0	0%	1%
APC	11	31%	3	4%	0	0%	11%
CDKN2A	0	0%	1	1%	2	18%	2%
ERBB2	1	3%	1	1%	0	0%	2%
FBXW7	4	11%	0	0%	0	0%	3%
GNA11	0	0%	1	1%	0	0%	1%
KIT	0	0%	1	1%	0	0%	1%
MAP2K1	0	0%	0	0%	1	9%	1%
PIK3CA	2	6%	1	1%	0	0%	2%
PTEN	2	6%	0	0%	0	0%	2%
SMAD4	5	14%	0	0%	1	9%	5%
TP53	12	33%	26	31%	4	36%	32%
total #	58		83		14		

### Clinical Reporting

The annotated variants obtained in VariantStudio were first filtered against intronic and synonymous variants with a VAF >5% and a total coverage at that position >300. The limited number of remaining variants was then classified according to the workflow presented in Fig 1. Only pathogenic or presumed pathogenic variants are reported. Each mutation is described separately with its clinical significance based on information obtained from My Cancer Genome, PCT MD Anderson and the literature [19–23]. Detailed information on the targeted panel and bio-informatics analysis is added. Samples that yield a mean coverage <300 are reported as non-informative, referring to the results of the orthogonal method or recommending a repeat analysis on another tumor sample. For informative samples (mean coverage >300), single amplicons that do not reach the minimal coverage threshold of 300 are listed in the report with a note that analysis of the particular hotspots was not feasible. Note that we also file variants with VAF between 3% and 5% if its coverage is high. These variants are also included in the report if the variant has well known pathogenicity and actionability, recommending however, a repeat analysis on another tumor sample. Our limit-of-detection assay (Fig 2) demonstrated that these are most likely correct as well.

## Discussion

As the number of actionable genes in solid tumors is steadily growing there is an increasing need to perform multi-gene mutation testing in molecular diagnostics. Several NGS panels including up to 75 genes are commercially available but these panels often contain genes or hotspots that are not of particular interest for molecular diagnostics due to their uncertain clinical significance, or to the lack of tumor types studied. We developed a custom panel to screen hotspots in 24 genes for clinically relevant mutations in NSCLC, CRC and melanoma, as well is in other solid tumor types. Our selection was based on information retrieved from COSMIC, My Cancer Genome, PCT MD Anderson and the available literature. Key factors determining our gene selection were 1) the present or likely future clinical significance in terms of therapeutic, prognostic or diagnostic value, both from clinical research and pre-clinical data; 2) the frequency of known hotspot mutations within the types of tumors predominantly screened in the hospital; and 3) the cost per sample, taking into account the number of samples weekly screened and the desired depth of coverage to allow for sufficient sensitivity. We found that the resulting total size of +/- 8 kb is a feasible compromise, offering a sufficiently extensive and clinically relevant mutational profiling in a cost-efficient way.

We have chosen for the TruSeq targeted enrichment method as it constitutes an easy workflow with acceptable hands-on time and excellent library normalization in combination with robust MiSeq instrumentation. This benchtop sequencer was selected for medium throughput analysis of about 15 tumor samples per week. It has been successfully implemented by several other diagnostic centers [10,19,20,24].

There are yet no fixed criteria for the validation of an NGS-based assay in molecular diagnostics. Approval for clinical accreditation is mainly based on guidelines set out by several authorized organizations. However, due to the large variability of the assay set-up, size, content, method, scope and instrumentation, the application of these guidelines can vary substantially making the interpretation of a consistent and reliable assay performance difficult and subjective. Here, we describe the validation of a custom targeted panel for solid tumors using TruSeq enrichment and sequencing of a pool of 12 libraries on a micro flow cell, with automatic data analysis with MiSeqReporter and further annotation and classification in VariantStudio. Newly encountered variants are always inspected in IGV. This study was conducted using NSCLC (n = 113), CRC (n = 76) and MELA cases (n = 16) as the primary aim was to replace the conventional methods for *EGFR*, *KRAS*, *NRAS* and *BRAF* mutation analysis.

In contrast to other validation studies that report the requirement of an initial qPCR quality control step to determine which samples should be excluded for NGS analysis, we do not discard any sample *a priori*. We have shown that tumor samples with a poor QC value still can yield acceptable minimal coverages at the requested hotspot positions allowing their diagnostic interpretation. This finding could be attributed to the rather short (150–180 bp) amplicons that are generated with the custom TruSeq method relative to the longer amplicon sizes used by other methods [25], in combination with the use of freshly embedded tissue samples. As a plus, removal of the QC qPCR step decreases the turnaround time with one full day in our workflow in which deparaffinization, tissue scraping and DNA extraction is performed on day 1 with the capture starting at day 2 and ending with running the libraries on the MiSeq. Finally, variant annotation and classification in VariantStudio is done at day 3.

Though it is difficult to compare targeted NGS studies because of the many variables, our sample drop-out rate of 13% (based on a mean coverage <300) is very similar or lower than those reported by others [10,19,20] even though we did not include the QC step. Our failed specimens were mainly from small lung biopsies or cytological preparations. The switch to the recently released TruSeq low input protocol is expected to decrease this rate substantially. In any case, the standard orthogonal methods should currently remain in place for the analysis of failed samples.

For the 55 FFPE samples analyzed in the validation study, no false negative or false positive variants were found, when the minimal mean coverage and the cut-off for VAF were set at 300 and 5%, respectively. Using these thresholds, all expected variants could be correctly demonstrated. Dilution series confirmed that variants could be detected even below the 3% allele frequency but to be on the safe side, we have set our threshold at 5%. This arbitrarily set VAF is used by several diagnostic laboratories. Actually, it also remains to be investigated what the biological or clinical significance is of (subclonal) mutations with a true low VAF, not caused by a low percentage of neoplastic cells in the starting material, which should always be taken into account. In this respect, especially for samples with low neoplastic cell contents (10 à 20%), actionable variants with a VAF between 3% and 5% and a high coverage are also considered as true but a repeat analysis on another tumor sample is recommended. The repeatability and reproducibility were maximal (100%), with high robustness of the entire workflow including data analysis. Finally, quality control testing of a Horizon reference sample (accuracy) identified all mutations at about the reported frequencies down to 2%. To summarize, our validation was performed on 55 FFPE samples in 8 runs each with 12 pooled libraries on a micro flow cell as summarized in Table 3 and S2 Table. The performance was demonstrated for SNVs as well as for indels.

**Table 3:** Summary of the validation assays and results for the targeted NGS screening on solid tumors (NSCLC, CRC and MELA).

Parameter	Samples	Conclusion	Data
Repeatability	6 FFPE	100% at >5% VAF	S4 Table
(intrarun)	(4 dupl, 2 tripl)		
Reproducibility	6 FFPE	100% at >5% VAF	S5 Table
(interrun)	(4 dupl, 2 tripl)		
Reproducibility	4 FFPE	100% at >5% VAF	S6 Table
(interoperator)	(4 dupl)		
Accuracy	Multiplex reference	100% at >1% VAF	Table 1
Specificity/	40 prospective	100% at >5% VAF	S7 Table
Sensitivity	FFPE	100% at >5% VAF	
Limit-of-detection	4 FFPE	down to at least 3% VAF	Fig 2
	Multiplex reference	down to at least 2% VAF	Table 1

Classification of variants remains a challenging task especially for those variants for which no or restricted information is available in databases, the literature, or on dedicated websites. We used the conservative approach so that variants with no proven link with tumorigenesis are classified as ‘variant of unknown significance’. We classify mutations as being ‘pathogenic’, when there is sufficient evidence for a role in cancer, and ‘presumed pathogenic’ where an association can clearly be expected, such as loss of function mutations in tumor suppressor genes (see Fig 1). The clinical relevance in terms of therapeutic, diagnostic and prognostic meaning is discussed within the interpretation section of the report, with reference to databases providing extensive insight in the levels of clinical evidence (for instance PCT MD Anderson). However, classifying variants in terms of both pathogenicity and clinical relevance often remains a rather subjective task and thus should best be performed in a clinical molecular tumor board where expertise is available from pathologists, oncologists, clinical biologists, bioinformaticians, and NGS scientists [26]. In any case, the community of molecular diagnostic oncology would benefit significantly from a standard scheme of classes as proposed by others [27], from guidelines how to classify variants, and from a curated classification database. Initial efforts towards these goals have recently been reported including a variant annotation scheme [28] and a database of potentially actionable cancer mutations [29].

Screening of a further set of 150 consecutive diagnostic samples revealed mutations in 86% of the 36 CRC specimens that could be analyzed, predominantly in *KRAS* (42%), *TP53* (33%), *APC* (31%), *BRAF* (14%), *SMAD4* (14%) and *FBXW7* (11%). In the 83 analyzed NSCLC specimens, clinically relevant variants were found in 73% with the highest contribution of *KRAS* (33%) and *TP53* (31%) followed by *EGFR* (12%) and *BRAF* (10%). Finally, in the 11 melanoma samples, *TP53* (36%), *BRAF* (27%), *NRAS* (27%) and *CDKN2A* (18%) were most abundantly detected. Overall, our data are in good agreement with those reported by others [20,30–35]. Differences between studies however, can be explained by the small sample sizes, the inclusion of different tumor subtypes, the content of the targeted panel, and/or a different classification scheme. These data show that the content of the panel allows for the detection of actionable variants that are presently relevant for NSCLC, CRC and MELA, replacing the conventional “gene-by-gene” analysis, but also of (1) variants that might predict sensitivity to drug classes that are being tested in clinical trials (e.g MEK-inhibitors or BRAF-inhibitors in respectively *KRAS*- and *BRAF*-mutated NSCLC, representing respectively 33% and 10% of the analyzed NSCLC cases) allowing for the selection of patients for these trials; (2) variants of genes other than the targeted genes that might mediate resistance to approved drugs (e.g. *PIK3CA* or *BRAF* mutations for anti-EGFR therapy); or (3) actionable variants in tumor types not used in this study (e.g. *KIT* and *PDGFRA* mutations in Gastro-Intestinal Stromal Tumors). In addition, combinations of detected variants might also help to identify the origin of a Neoplasia of Unknown Primary (NUP) (eg. *APC* and *KRAS* mutations in NUP suggesting a colorectal origin). Further validation of this custom targeted panel as a pan-solid tumor panel for all these additional indications is ongoing.

In conclusion, this validation study demonstrated the effectiveness of solid tumor NGS screening for the detection of mutations with prognostic and therapeutic value using a custom-designed targeted panel on the MiSeq. We also showed that sequencing all samples, irrespective of their QC, provides a very similar fall-out rate as for the reported preselected cohorts of samples thus generating a higher diagnostic yield. We have now implemented this assay in the routine diagnostics for NSCLC, CRC and malignant melanoma and in a validation setting for several other solid tumor types, as the panel content includes targets that are significantly mutated and relevant in many other tumor types.

## Supporting Information

S1 Fig Start amount of DNA vs mean coverage. (PPTX)

S2 Fig Estimated tumor zone size vs mean coverage. (PPTX)

S1 Table Content of custom targeted solid tumor panel. (DOCX)

S2 Table Overview of FFPE samples used for the validation. (DOCX)

S3 Table Example of the sample coverage file. (XLSX)

S4 Table Intrarun repeatability. (DOCX)

S5 Table Interrun reproducibility. (DOCX)

S6 Table Interoperator reproducibility. (DOCX)

S7 Table Specificity assay. (DOCX)

S8 Table Run QC values. (DOCX)
