# Supplementary material for: Validation and Application of a Custom-Designed Targeted Next-Generation Sequencing Panel for the Diagnostic Mutational Profiling of Solid Tumors
Source: PLoS One. 2016 Apr 21;11(4):e0154038. doi: 10.1371/journal.pone.0154038 (PMC4839685; doi:10.1371/journal.pone.0154038)
Supplement: S1 Fig — (PPTX) [file pone.0154038.s001.pptx]

## Slide 1
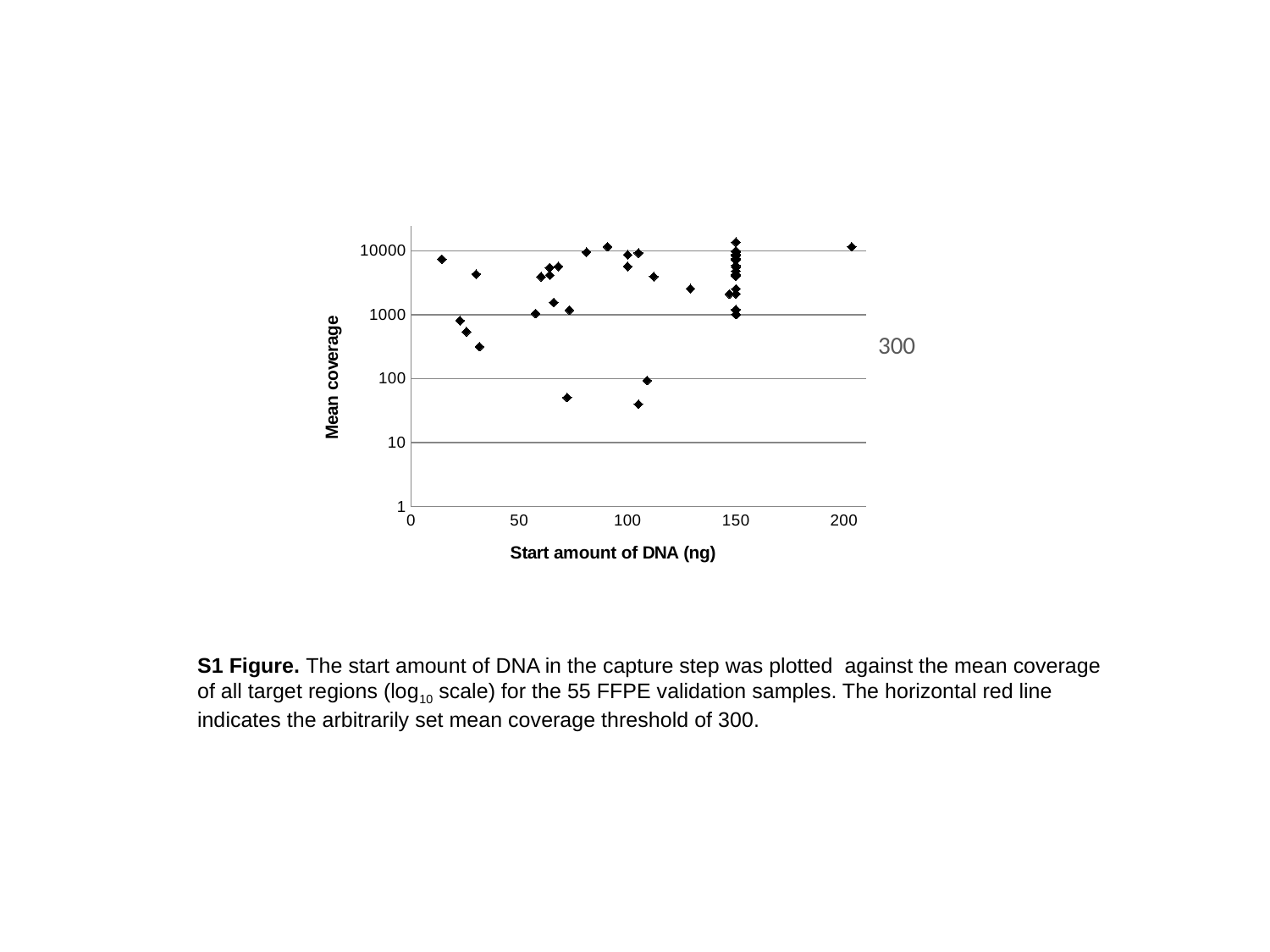

### Chart
| Category | | meancov |
|---|---|---|300
S1 Figure. The start amount of DNA in the capture step was plotted against the mean coverage of all target regions (log10 scale) for the 55 FFPE validation samples. The horizontal red line indicates the arbitrarily set mean coverage threshold of 300.
