# Supplementary material for: Validation and Application of a Custom-Designed Targeted Next-Generation Sequencing Panel for the Diagnostic Mutational Profiling of Solid Tumors
Source: PLoS One. 2016 Apr 21;11(4):e0154038. doi: 10.1371/journal.pone.0154038 (PMC4839685; doi:10.1371/journal.pone.0154038)
Supplement: S2 Fig — (PPTX) [file pone.0154038.s002.pptx]

## Slide 1
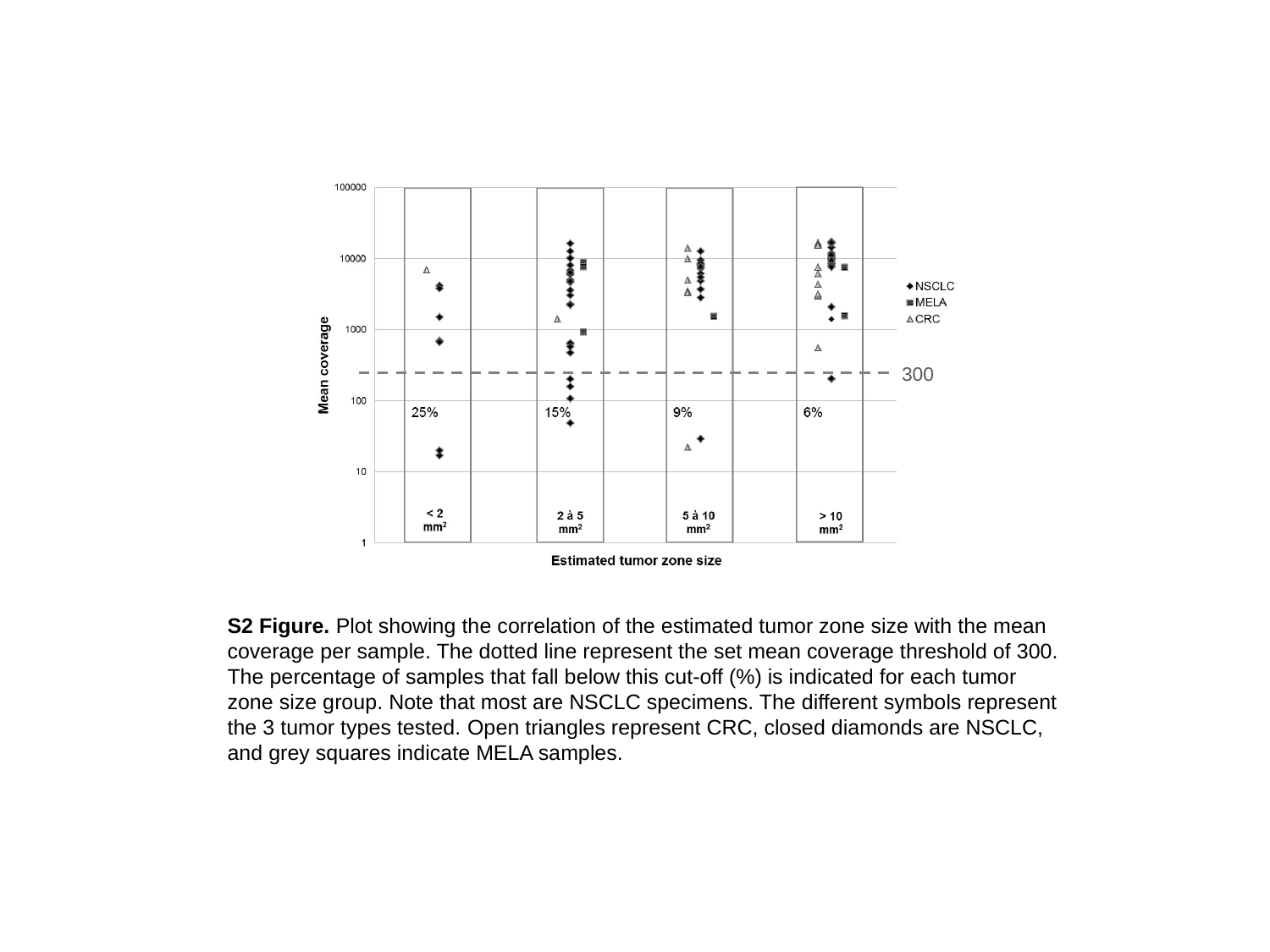

300
S2 Figure. Plot showing the correlation of the estimated tumor zone size with the mean coverage per sample. The dotted line represent the set mean coverage threshold of 300. The percentage of samples that fall below this cut-off (%) is indicated for each tumor zone size group. Note that most are NSCLC specimens. The different symbols represent the 3 tumor types tested. Open triangles represent CRC, closed diamonds are NSCLC, and grey squares indicate MELA samples.
