# Supplementary material for: Validation and Application of a Custom-Designed Targeted Next-Generation Sequencing Panel for the Diagnostic Mutational Profiling of Solid Tumors
Source: PLoS One. 2016 Apr 21;11(4):e0154038. doi: 10.1371/journal.pone.0154038 (PMC4839685; doi:10.1371/journal.pone.0154038)
Supplement: S1 Table — (DOCX) [file pone.0154038.s003.docx]

**S1 Table.** Content of the custom solid targeted tumor panel. Gene names are provided with their classification as a tumor suppressor gene (Ts) or not, and the Genbank transcript ID used here. In the Hs0 to Hs6 columns, the different hotspots within these genes are provided, indicated with the one-letter amino acid code and its position in the protein. In tumor suppressor genes, all stopgain (stop) and frameshift (fs) variants resulting in loss-of-function mutations are regarded as detrimental. Hs: Hotspot; ex: exon; del: deletion; ins: insertion.

| **Gene** | **Ts** | **NM_ID** |  | **Hs0** | **Hs1** | | **Hs2** | | **Hs3** | | **Hs4** | **Hs5** | **Hs6** |
| --- | --- | --- | --- | --- | --- | --- | --- | --- | --- | --- | --- | --- | --- |
| *AKT1* |  | NM_001014431 |  | E17 |  | |  | |  | |  |  |  |
| *ALK* |  | NM_004304 |  | F1174 | R1275 | |  | |  | |  |  |  |
| *APC* | Y | NM_000038 |  | stop & fs |  |  | |  | |  |  |  |  |
| *BRAF* |  | NM_004333 |  | G466 | G469 | | V600 | |  | |  |  |  |
| *CDKN2A* | Y | NM_001195132 |  | stop & fs | P114 |  | |  | |  |  |  |  |
| *CTNNB1* | Y | NM_001098210 |  | stop & fs | D32 | | S33 | | G34 | | S37 | T41 | S45 |
| *EGFR* |  | NM_005228 |  | G719 | S768 | | ex19del | | ex20ins | | T790 | L858 | L861 |
| *ERBB2* |  | NM_004448 |  | S310 | L755 | |  | |  | |  |  |  |
| *FBXW7* | Y | NM_033632 |  | stop & fs | R465 | | R479 | | R505 | |  |  |  |
| *FGFR2* |  | NM_022970 |  | S252 | N549 | |  | |  | |  |  |  |
| *GNA11* |  | NM_002067 |  | Q209 |  | |  | |  | |  |  |  |
| *GNAQ* |  | NM_002072 |  | Q209 |  | |  | |  | |  |  |  |
| *KIT* |  | NM_000222 |  | W557 | V559 | | V560 | | L576 | | D816 | N822 |  |
| *KRAS* |  | NM_033360 |  | G12 | G13 | | A59 | | Q61 | | K117 | A146 |  |
| *MAP2K1* |  | NM_002755 |  | K57 | P124 | |  | |  | |  |  |  |
| *MET* |  | NM_001127500 |  | T1010 | Y1253 | |  | |  | |  |  |  |
| *NRAS* |  | NM_002524 |  | G12 | G13 | | A59 | | Q61 | | K117 | A146 |  |
| *PDGFRA* |  | NM_006206 |  | D842 |  | |  | |  | |  |  |  |
| *PIK3CA* |  | NM_006218 |  | E542 | E545 | | H1047 | |  | |  |  |  |
| *PTEN* | Y | NM_000314 |  | stop & fs | R130 | | R173 | |  | |  |  |  |
| *RET* |  | NM_020975 |  | C634 | M918 | |  | |  | |  |  |  |
| *SMAD4* | Y | NM_005359 |  | stop & fs | R361 | |  | |  | |  |  |  |
| *STK11* | Y | NM_000455 |  | stop & fs | F354 | |  | |  | |  |  |  |
| *TP53* | Y | NM_000546 |  | stop & fs | R175 | | R248 | | R273 | |  |  |  |
