# Supplementary material for: Validation and Application of a Custom-Designed Targeted Next-Generation Sequencing Panel for the Diagnostic Mutational Profiling of Solid Tumors
Source: PLoS One. 2016 Apr 21;11(4):e0154038. doi: 10.1371/journal.pone.0154038 (PMC4839685; doi:10.1371/journal.pone.0154038)
Supplement: S2 Table — (DOCX) [file pone.0154038.s004.docx]

**S2 Table.** Overview of the 55 FFPE samples (14 NSCLC, 36 CRC, 5 MELA) used for the different validation assays. The retrospective samples with known mutations are indicated in bold. The ‘Accuracy’ was tested on the Multiplex Reference sample (Horizon Dx) but the NGS data from all FFPE validation samples could be included as well. ‘%’ indicates the percentage of tumor cells in the sample.

|  |  |  | **1. Intrarun** | **2. Interrun** | **3. Interoperator** | **5. Sensit/Specif** | **6. Limit-of-detect** |  |  |  |  |  | **1. Intrarun** | **2. Interrun** | **3. Interoperator** | **5. Sensit/Specif** | **6. Limit-of-detect** |
| --- | --- | --- | --- | --- | --- | --- | --- | --- | --- | --- | --- | --- | --- | --- | --- | --- | --- |
| **N°** | **Sample ID** | **%** |  |  |  |  |  |  |  | **N°** | **Sample ID** | **%** |  |  |  |  |  |
| 1 | NSCLC1 | 20% | Y |  |  | Y |  |  |  | 29 | CRC15 | 50% |  |  |  | Y |  |
| 2 | NSCLC2 | 70% | Y |  |  | Y |  |  |  | 30 | CRC16 | 60% |  |  |  | Y |  |
| 3 | **NSCLC3** | 40% | **Y** |  |  |  |  |  |  | 31 | CRC17 | 60% |  |  |  | Y |  |
| 4 | NSCLC4 | 40% |  | Y |  | Y |  |  |  | 32 | CRC18 | 50% |  |  |  | Y |  |
| 5 | **NSCLC5** | 40% |  |  | **Y** |  |  |  |  | 33 | CRC19 | 60% |  |  |  | Y |  |
| 6 | NSCLC6 | 40% |  |  |  | Y |  |  |  | 34 | CRC20 | 60% |  |  |  | Y |  |
| 7 | NSCLC7 | 40% |  |  |  | Y |  |  |  | 35 | CRC21 | 70% |  |  |  | Y |  |
| 8 | NSCLC8 | 60% |  |  |  | Y |  |  |  | 36 | CRC22 | 60% |  |  |  | Y |  |
| 9 | NSCLC9 | 60% |  |  |  | Y |  |  |  | 37 | CRC23 | 70% |  |  |  | Y |  |
| 10 | NSCLC10 | 20% |  |  |  | Y |  |  |  | 38 | CRC24 | 50% |  |  |  | Y |  |
| 11 | NSCLC11 | 40% |  |  |  | Y |  |  |  | 39 | CRC25 | 50% |  |  |  | Y |  |
| 12 | NSCLC12 | 60% |  |  |  | Y |  |  |  | 40 | CRC26 | 40% |  |  |  | Y |  |
| 13 | NSCLC13 | 50% |  |  |  | Y |  |  |  | 41 | CRC27 | 50% |  |  |  | Y |  |
| 14 | NSCLC14 | 60% |  |  |  |  | **Y** |  |  | 42 | CRC28 | 70% |  |  |  | Y |  |
| 15 | CRC1 | 60% | Y |  |  | Y |  |  |  | 43 | CRC29 | 50% |  |  |  | Y |  |
| 16 | **CRC5** | 40% | **Y** |  |  |  |  |  |  | 44 | CRC30 | 60% |  |  |  | Y |  |
| 17 | **CRC3** | 30% |  | **Y** |  |  |  |  |  | 45 | CRC31 | 50% |  |  |  | Y |  |
| 18 | **CRC4** | 60% |  | **Y** |  |  |  |  |  | 46 | CRC32 | 50% |  |  |  | Y |  |
| 19 | **CRC5** | 40% |  | **Y** |  |  |  |  |  | 47 | CRC33 | 40% |  |  |  | Y |  |
| 20 | **CRC6** | 80% |  | **Y** | **Y** |  |  |  |  | 48 | CRC34 | 40% |  |  |  | Y |  |
| 21 | **CRC7** | 40% |  |  | **Y** |  |  |  |  | 49 | **CRC35** | 50% |  |  |  |  | **Y** |
| 22 | **CRC8** | 30% |  |  | **Y** |  |  |  |  | 50 | CRC36 | 70% |  |  |  |  | **Y** |
| 23 | CRC9 | 40% |  |  |  | Y |  |  |  | 51 | **MELA1** | 40% | **Y** |  |  |  |  |
| 24 | CRC10 | 40% |  |  |  | Y |  |  |  | 52 | **MELA2** | 80% |  | **Y** |  |  |  |
| 25 | CRC11 | 40% |  |  |  | Y |  |  |  | 53 | MELA3 | 70% |  |  |  | Y |  |
| 26 | CRC12 | 30% |  |  |  | Y |  |  |  | 54 | MELA4 | 70% |  |  |  | Y |  |
| 27 | CRC13 | 30% |  |  |  | Y |  |  |  | 55 | **MELA5** | 70% |  |  |  |  | **Y** |
| 28 | CRC14 | 30% |  |  |  | Y |  |  |  |  | total |  | 6 | 6 | 4 | 40 | 4 |
