# Supplementary material for: Validation and Application of a Custom-Designed Targeted Next-Generation Sequencing Panel for the Diagnostic Mutational Profiling of Solid Tumors
Source: PLoS One. 2016 Apr 21;11(4):e0154038. doi: 10.1371/journal.pone.0154038 (PMC4839685; doi:10.1371/journal.pone.0154038)
Supplement: S5 Table — (DOCX) [file pone.0154038.s007.docx]

**S5 Table.** Interrun reproducibility tested in different runs on 6 FFPE samples from different tumor origin (NSCLC, CRC or MELA) and with variable tumor percentages (%). Gene mutations (Mut) are shown with their allele frequencies (VAF) and coverages (Cov). The mean VAF and standard deviation (SD) of each mutation is also provided. Runs were always performed with 12 pooled samples. ins: insertion.

| **N°** | **Sample** | **%** |  | **Gene1** | **Mut** | **VAF** | **Cov** |  | **mean** | **SD** |  | **Gene2** | **Mut** | **VAF** | **Cov** |  | **mean** | **SD** |
| --- | --- | --- | --- | --- | --- | --- | --- | --- | --- | --- | --- | --- | --- | --- | --- | --- | --- | --- |
| 1 | NSCLC4 | 40 |  | TP53 | P278R | 21% | 1723 |  | 20% | 1% |  |  |  |  |  |  |  |  |
|  | NSCLC4 | 40 |  | TP53 | P278R | 20% | 1293 |  |  |  |  |  |  |  |  |  |  |  |
| 2 | CRC3 | 30 |  | KRAS | G12V | 24% | 2792 |  | 26% | 3% |  |  |  |  |  |  |  |  |
|  | CRC3 | 30 |  | KRAS | G12V | 28% | 218 |  |  |  |  |  |  |  |  |  |  |  |
| 3 | CRC4 | 60 |  | NRAS | Q61L | 48% | 811 |  | 49% | 1% |  |  |  |  |  |  |  |  |
|  | CRC4 | 60 |  | NRAS | Q61L | 50% | 2893 |  |  |  |  |  |  |  |  |  |  |  |
| 4 | MELA2 | 80 |  | NRAS | Q61R | 47% | 1992 |  | 40% | 10% |  |  |  |  |  |  |  |  |
|  | MELA2 | 80 |  | NRAS | Q61R | 33% | 306 |  |  |  |  |  |  |  |  |  |  |  |
|  |  |  |  |  |  |  |  |  |  |  |  |  |  |  |  |  |  |  |
| 5 | CRC5 | 40 |  | KRAS | A146V | 41% | 31330 |  | 40% | 2% |  | PTEN | D268ins1bp | 29% | 11333 |  | 27% | 2% |
|  | CRC5 | 40 |  | KRAS | A146V | 38% | 14809 |  |  |  |  | PTEN | D268ins1bp | 27% | 8574 |  |  |  |
|  | CRC5 | 40 |  | KRAS | A146V | 41% | 29592 |  |  |  |  | PTEN | D268ins1bp | 26% | 14554 |  |  |  |
| 6 | CRC6 | 80 |  | KRAS | G12C | 13% | 3722 |  | 17% | 3% |  | TP53 | M237I | 23% | 12689 |  | 22% | 2% |
|  | CRC6 | 80 |  | KRAS | G12C | 20% | 603 |  |  |  |  | TP53 | M237I | 20% | 860 |  |  |  |
|  | CRC6 | 80 |  | KRAS | G12C | 18% | 3679 |  |  |  |  | TP53 | M237I | 22% | 8118 |  |  |  |
