# Supplementary material for: Validation and Application of a Custom-Designed Targeted Next-Generation Sequencing Panel for the Diagnostic Mutational Profiling of Solid Tumors
Source: PLoS One. 2016 Apr 21;11(4):e0154038. doi: 10.1371/journal.pone.0154038 (PMC4839685; doi:10.1371/journal.pone.0154038)
Supplement: S7 Table — (DOCX) [file pone.0154038.s009.docx]

**S7 Table.** Sensitivity and specificity assay on 40 prospective FFPE tumor samples that were analyzed by NGS and the standard assays. Only the 20 samples in which a hotspot mutation in *EGFR, KRAS, NRAS* or *BRAF* was detected by NGS in accordance with the acceptance criteria (coverage >300 and VAF >5%), are listed. All mutations were confirmed by the respective orthogonal assay, which did not detect any additional hotspot mutation in these 4 genes. %: percent of tumor cells.

| **N°** | **tumor type** | **%** |  | **Gene** | **Mut** | **VAF** | **Cov** |
| --- | --- | --- | --- | --- | --- | --- | --- |
| 1 | NSCLC1 | 20 |  | EGFR | ins20 | 11% | 671 |
| 2 | NSCLC2 | 70 |  | KRAS | G12D | 96% | 24000 |
| 3 | NSCLC6 | 80 |  | EGFR | L858R | 48% | 7597 |
| 4 | CRC1 | 60 |  | KRAS | G12S | 56% | 2292 |
| 5 | CRC9 | 40 |  | KRAS | G12V | 30% | 3655 |
| 6 | CRC12 | 30 |  | KRAS | G12V | 19% | 3266 |
| 7 | CRC13 | 30 |  | KRAS | G12V | 22% | 405 |
| 8 | CRC14 | 30 |  | KRAS | Q61H | 15% | 10758 |
| 9 | CRC16 | 60 |  | KRAS | A146V | 40% | 9516 |
| 10 | CRC17 | 60 |  | BRAF | V600E | 35% | 7282 |
| 11 | CRC18 | 50 |  | KRAS | G12V | 18% | 3013 |
| 12 | CRC19 | 60 |  | BRAF | V600E | 20% | 14306 |
| 13 | CRC21 | 70 |  | BRAF | V600E | 58% | 1551 |
| 14 | CRC23 | 70 |  | BRAF | V600E | 37% | 17898 |
| 15 | CRC24 | 50 |  | KRAS | G12S | 41% | 1645 |
| 16 | CRC31 | 50 |  | KRAS | A146T | 14% | 1832 |
| 17 | CRC32 | 50 |  | KRAS | G13D | 37% | 655 |
| 18 | CRC33 | 40 |  | NRAS | Q61R | 33% | 985 |
| 19 | CRC34 | 40 |  | BRAF | V600E | 26% | 2371 |
| 20 | MELA3 | 70 |  | BRAF | V600E | 18% | 13644 |
