# Supplementary material for: Validation and Application of a Custom-Designed Targeted Next-Generation Sequencing Panel for the Diagnostic Mutational Profiling of Solid Tumors
Source: PLoS One. 2016 Apr 21;11(4):e0154038. doi: 10.1371/journal.pone.0154038 (PMC4839685; doi:10.1371/journal.pone.0154038)
Supplement: S8 Table — (DOCX) [file pone.0154038.s010.docx]

**S8 Table.** For 10 subsequent diagnostic runs, the QC values of the monitored parameters are provided. Mean and standard deviations (SD) are shown for each QC parameter.

| **Run N°** |  | **Cluster density** | **Cluster Pass filter (%)** | **%≥Q30** | **Error rate (%)** |
| --- | --- | --- | --- | --- | --- |
| RunD1 |  | 933 | 94.8 | 93.3 | 0.60 |
| RunD2 |  | 561 | 98.2 | 97.4 | 0.61 |
| RunD3 |  | 882 | 96.0 | 96.0 | 0.64 |
| RunD4 |  | 1128 | 85.8 | 91.1 | 0.64 |
| RunD5 |  | 1510 | 87.5 | 86.8 | 0.76 |
| RunD6 |  | 1649 | 83.7 | 90.1 | 1.16 |
| RunD7 |  | 1312 | 91.1 | 93.0 | 0.71 |
| RunD8 |  | 944 | 98.3 | 93.2 | 0.54 |
| RunD9 |  | 632 | 97.6 | 96.0 | 0.62 |
| RunD10 |  | 899 | 95.9 | 89.1 | 0.61 |
| **mean** |  | **1045** | **92.9** | **92.6** | **0.69** |
| SD |  | 337 | 5.2 | 3.2 | 0.17 |
